# A single-subject study to evaluate the inhibitory repetitive transcranial magnetic stimulation combined with traditional dysphagia therapy in patients with post-stroke dysphagia

**Published:** 2016-07-06

**Authors:** Leila Ghelichi, Mohammad Taghi Joghataei, Shohreh Jalaie, Noureddin Nakhostin-Ansari, Bijan Forogh, Masoud Mehrpour

**Affiliations:** 1 Department of Speech and Language Pathology, School of Rehabilitation Sciences, Iran University of Medical Sciences, Tehran, Iran; 2 Cellular and Molecular Research Center, Department of Anatomy, School of Medicine, Iran University of Medical Sciences, Tehran, Iran; 3 Department of Physiotherapy, School of Rehabilitation, Tehran University of Medical Sciences, Tehran, Iran; 4 Department of Physical Medicine and Rehabilitation, School of Medicine, Iran University of Medical Sciences, Tehran, Iran; 5 Department of Neurology, Firouzgar Hospital, Iran University of Medical Sciences, Tehran, Iran

**Keywords:** Stroke, Dysphagia, Rehabilitation, Combined Modality Therapy, Transcranial Magnetic Stimulation, Deglutition Disorders

## Abstract

**Background:** Post-stroke dysphagia is common and is associated with the development of pneumonia. To investigate the effects of repetitive transcranial magnetic stimulation (rTMS) combined with traditional dysphagia therapy (TDT) on swallowing function in patients with post-stroke dysphagia.

**Methods:** In this single-subject study, four patients with dysphagia post-stroke included. The patients received the rTMS applied to the intact cerebral hemisphere at 1 Hz with train of 1200 for 5 consecutive days combined with TDT 3 days per week for 6 weeks. The main outcome measure was the Mann Assessment of Swallowing Ability (MASA). Measurements were taken before, after the end of 5^th^, 10^th^, 15^th^ treatment sessions, and after the end of the treatment (18^th^ session).

**Results:** The MASA scores improved in all patients following treatment. The maximum and minimum change in level between the baseline phase and treatment phase was +84 and +36. The greatest percentage improvement was observed after 5^th^ treatment sessions ranging between 11 and 35%. The treatment trend was upward shown by the directions of the slopes indicated by positive values (+9.1-+20.7). The dysphagia was resolved after 10^th^ treatment session in all participants. The aspiration resolved in two participants after the 5^th^ treatment session and resolved in another 2 participants after the 10^th^ treatment session.

**Conclusion:** The combination therapy of rTMS plus TDT improved swallowing function in patients with post-stroke dysphagia. Further research with a larger sample size is recommended.

## Introduction

Stroke is a leading cause of disability in adult population globally. It can cause various medical and neurological complications such as dysphagia or swallowing problem. Impaired swallowing is a common complication with the prevalence to be approximately between 42 and 67% after stroke.^1^^-^^3^ Stroke related dysphagia (SRD) as a neuromuscular disorder is important because it can affect the activities of daily living and quality of life of stroke survivors.

The SRD must be treated effectively because it is associated with mortality and increased length of hospital stay.^4^ The treatment of SRD include percutaneous endoscopic gastrostomy or nasogastric tube feeding,^5^ and rehabilitation techniques of sensory enhancement techniques,^6^^,^^7^ functional dysphagia therapy,^5^ exercise therapy (e.g. Lee Silverman Voice Treatment and the Shaker Head Lift),^8^ and compensatory treatment procedures.^9^^,^^10^

Recently, the repetitive transcranial magnetic stimulation (rTMS) has been used to treat post-stroke dysphagia.^11^^-^^15^ However, the authors have reported that the rTMS must be used with rehabilitation techniques to be sufficiently effective.^16^ To investigate the safety and feasibility of rTMS combined with swallowing rehabilitation for post-stroke dysphagia, Momosaki et al.^16^ treated 4 patients with post-stroke dysphagia with rTMS at 3 Hz applied to the pharyngeal motor cortex bilaterally combined with 20 minutes of swallowing rehabilitation exercises and concluded that the protocol of rTMS plus swallowing rehabilitation exercise seems to be safe and feasible for patients with SRD.

The effects of rTMS plus swallowing rehabilitation treatments are not evaluated in patients with SRD. Therefore, the aim of this study was to investigate the effects of rTMS combined with traditional dysphagia therapy (TDT) in patients with post-stroke dysphagia.

## Materials and Methods

This study used an A-B single-subject design with measurements taken on four patients suffering from SRD. The Ethical Committee of Tehran University of Medical Sciences, Iran, approved the study, and all patients gave their written informed consent.

In this study, the Mann Assessment of Swallowing Ability (MASA) was used as the main outcome measure. 

Four patients with SRD included in the study. The inclusion criteria were (1) age ≥ 18 years old and (2) first-ever stroke resulted in dysphagia. The patients excluded if they had (1) dementia, (2) other neurological diseases, and (3) history of recurrent stroke.

Patients underwent a baseline interview to collect the demographic data by a speech-language pathologist (SLP). The MASA^17^ was administered to assess dysphagia before, after the end of 5^th^, 10^th^, 15^th^ sessions, and finally after the end of treatment. Therefore, 1 assessment was performed pre-treatment and 4 assessments were carried out during the treatment phase. Then, the patients received traditional treatment for 6 weeks, 3 days a week combined with rTMS (every day for 5 consecutive days). 

The MASA is a simple to use instrument, which has been reported to be reliable and valid to document the swallowing function in patients with stroke.^17^^,^^18^ This is a 24 clinical item tool arranged from the preparatory oral phase to pharyngeal phase and is comprised 3 components of swallowing: (1) oral motor/sensory, (2) functionality, and (3) recommendations for dietary.^17^^,^^18^ The MASA scoring system includes a total score out of 200, and an ordinal score for both dysphagia (nil ≤ 178-200; mild ≤ 168-177; moderate ≤ 139-167; and severe ≤ 138) and aspiration (nil ≤ 170-200; mild ≤ 149-169; moderate ≤ 148; and severe ≤ 140).

Treatment consisting of rTMS (5 sessions) combined with TDT were given to each patient. The rehabilitation exercises of TDT included 30 minutes individualized oral motor exercises, swallowing maneuvers, compensatory strategies, and sensory stimuli, 3 days per week for 6 weeks. Table 1 shows the detail of the swallowing exercises provided by a SLP.

The magstim super-rapid stimulator (Magstim, Whitland, Dyfed, UK) and a figure-of-eight coil (Whitland, Dyfed, UK) were used for our low-frequency rTMS protocol. The inhibitory rTMS procedure was targeted the intact cerebral hemisphere with a train of 1200 pulses at 1 Hz, with stimulus strength at 20% above the resting motor threshold for 20 minutes.

**Table 1:** Traditional dysphagia therapy (TDT)

**Type of traditional treatment**	**Examples/Description**
Exercise programs	Oral motor control exercises
	Range of motion tongue exercises
	Resistance exercises
	Bolus control exercises
	Bolus propulsion exercises
	Laryngeal elevation
	Shaker exercises
Pharyngeal swallowing maneuvers	Mendelsohn maneuver
	Supraglottic swallow
	Super supraglottic swallow
	Effortful swallow
	Masako maneuver
Compensatory swallowing strategies	Slow rate
	Small bites and sips
	Viscosity changes to food and liquids
	Positional changes (up right, chin tuck, head rotation, head tilt)
	Clear throat or cough after each bite/sip
	No straws
	Place food on right or left side of mouth
	Alternate bite/sip
Sensory stimuli	Changing the taste, volume, temperature, or carbonation of the bolus
	Thermal tactile stimulation
	Additional pressure on the tongue with a spoon

The optimal point of stimulation was located where the maximum motor evoked potentials (MEP) were obtained for the mylohyoid muscles.^15^ The EMG machine (EL258RT, Biopac, Santa Barbara, CA, USA) was used to record MEP using two pairs of shielded bipolar silver-silver chloride surface electrodes was used with bandpass filter at 2-5 kHz, frequency at 20 kHz, and sweep speed at 1 second. A physiatrist applied the rTMS considering the safety recommendations and guidelines.^19^ The week 1 treatment protocol included 5 rTMS treatments applied daily plus 3 TDT every other day.

The visual analysis was used for interpreting data. The level was calculated for the differences between the baseline phase and treatment phase data. To quantify the trend, slopes were computed.

## Results

Four patients with dysphagia (all male; age range 59-72 years) included and completed the combination therapy. The time between stroke onset and intervention ranged from 1 to 18 months (Table 2).

As shown in table 3, the MASA scores improved in all patients following treatment. The percentage improvement after 5^th^ treatment session was ranged between 11 and 35%.

Participant 1 (26%) and patient 3 (35%), both with subcortical stroke, showed the greatest percentage improvement at this stage. Participants 2 and 4, both with cortex stroke, improved 11% after the 5^th^ treatment session.

**Table 2:** Demographic characteristics of participants

**Participant**	**Age (year)**	**Stroke localization**	**Time stroke onset to intervention (month)**
1	59	Subcortical	6
2	60	Cortex	18
3	70	Subcortical	1
4	72	Cortex	10
Mean ± SD	65.2 ± 6.7	-	8.7 ± 7.2

SD: Standard deviation

**Table 3:** Mann Assessment of Swallowing Ability (MASA) score, severity of dysphagia and severity of aspiration, pre- and post-treatment

**Patients**	**Patient 1**	**Patient 2**	**Patient 3**	**Patient 4**
MASA score				
Pre-treatment	119	160	108	153
Fifth session	150	175	146	170
10^th^ session	179	185	178	180
15^th^ session	185	194	185	190
18^th^ session	194	196	192	194
Dysphagia				
Pre-treatment	Yes-Severe	Yes-Moderate	Yes-Severe	Yes-Moderate
Fifth session	Yes-Moderate	Yes-Mild	Yes-Moderate	Yes-Mild
10^th^ session	No	No	No	No
15^th^ session	No	No	No	No
18^th^ session	No	No	No	No
Aspiration				
Pre-treatment	Yes-Severe	Yes-Mild	Yes-Severe	Yes-Mild
Fifth session	Yes-Mild	No	Yes-Moderate	No
10^th^ session	No	No	No	No
15^th^ session	No	No	No	No
18^th^ session	No	No	No	No

MASA: Mann Assessment of Swallowing Ability

The percentage improvement after 10^th^ treatment session compared to the 5^th^ treatment session was 4-22%. Again, the greatest improvement was observed in participants 1 (19%) and 3 (22%). Participants 2 and 4 showed 4 and 6% improvement, respectively, after the 10^th^ treatment session (Table 3).

**Figure 1:**
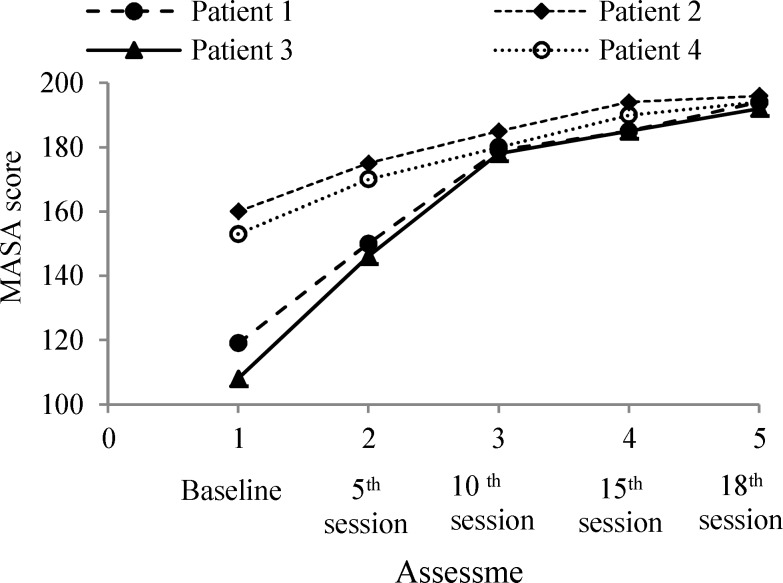
Trend was upward after combined therapy (Slopes values: participant 1: +18.5, participant 2: +9.1, participant 3: +20.7, and participant 4: +10.2)

The maximum and minimum change in level between the baseline phase and treatment phase was +84 and +36 observed in participants 3 and 2, respectively. The treatment trend was upward as shown by the directions of the slopes indicated by positive values (+9.1-+20.7) (Figure 1).

MASA: Mann Assessment of Swallowing Ability

According to the MASA ordinal scores, the severity of both dysphagia and aspiration improved during the treatment phase. The dysphagia was resolved after 10^th ^treatment session in all participants. The aspiration, however, resolved in participants 2 and 4 after 5^th^ treatment session (cortex stroke) but resolved in participants 1 and 3 (subcortical stroke) after 10^th^ treatment session (Table 3).

## Discussion

The results of this single-subject study support the usefulness of combination therapy of rTMS plus TDT for post-stroke dysphagia. The findings of the present study in accordance with Momosaki et al.^16^ showed that the combination therapy protocol is safe, feasible, and effective for the treatment of patients with SRD. 

The results demonstrated that the patients improved on MASA scores indicating improvements in dysphagia and aspiration post-stroke following rTMS plus TDT protocol.

The greatest improvements were obtained during the first five treatment session where patients received rTMS in combination with TDT. The greatest improvements after 5^th^ treatment session exhibited by patients imply that the addition of rTMS to the TDT had beneficial effects on patients’ outcome. 

Participants with subcortical stroke who had the lowest scores on MASA pretreatment showed the greatest improvements after 5^th^ treatment session. This finding indicates that the patients with initially more severe dysphagia may benefit more from combination therapy.

Although patients with initially low scores on MASA exhibited more improvements after 5^th^ treatment session, it is important to note that patients with chronic, cortex stroke (participants 2 and 4) who had initially better scores on MASA achieved complete improvement of aspiration after 5^th^ treatment session. Depending on the severity of the dysphagia post-stroke, however, patients may need more treatment sessions to resolve both the dysphagia and aspiration. Even though, the improvements were found after the end of the treatment (18^th^ treatment session), all participants improved completely after 10^th^ treatment session. This indicates that the 10 treatment sessions of combination therapy may be sufficient to resolve the dysphagia as well as aspiration after stroke. This finding needs to be confirmed with more investigations using high-quality designs with a large sample of patients.

In this study, rTMS was delivered unilaterally to the intact cerebral hemisphere combined with TDT, which resulted in the significant improvement of post-stroke dysphagia.

The improvement may be explained by the inhibitory effects of rTMS that could give rise to the decrease in transcallosal inhibition from the intact hemisphere to the damaged one; this effect might modulate a neural network of cortex associated with the swallowing function. 

Previous reports using bilateral rTMS found improvement in dysphagia, as well.^12^^,^^16^ The improvements obtained in these cases could be also due to the exercises performed using a traditional therapy that has been shown to increase lingual strength with improvements in swallowing function in patients with acute and chronic post-stroke dysphagic.^20^ It is possible that the combination effects from rTMS and TDT induced neural plasticity that translated into the improvements in swallowing function. A study is needed to compare the effects of combination therapy with rTMS or TDT. 

## Conclusion

The results from this single-subject study suggest that that the combination of rTMS plus TDT improved swallowing function in patients with post-stroke dysphagia. This finding that participants with post-stroke dysphagia benefitted from rTMS plus TDT are important because it provides the therapist with a treatment method that is not only effective but also it may improve the post-stroke dysphagia quite quickly. Further research with a larger sample size is needed to confirm the findings.

## Notes:

**How to cite this article:** Ghelichi L, Joghataei MT, Jalaie S, Nakhostin-Ansari N, Forogh B, Mehrpour M. A single-subject study to evaluate the inhibitory repetitive transcranial magnetic stimulation combined with traditional dysphagia therapy in patients with post-stroke dysphagia. Iran J Neurol 2016; 15(3): 140-5.
